# Differential Sorting of Microparticles Using Spiral Microchannels with Elliptic Configurations

**DOI:** 10.3390/mi11040412

**Published:** 2020-04-14

**Authors:** Kaan Erdem, Vahid Ebrahimpour Ahmadi, Ali Kosar, Lütfullah Kuddusi

**Affiliations:** 1 Mechanical Engineering Program, Graduate School of Science Engineering and Technology, Istanbul Technical University, Maslak, 34496 Istanbul, Turkey; erdemk@itu.edu.tr; 2 Mechanical Engineering Department, Faculty of Engineering and Natural Sciences, Istanbul Medeniyet University, Uskudar, 34700 Istanbul, Turkey; 3 Sabanci University Nanotechnology Research Center (SUNUM), Sabanci University, Tuzla, 34956 Istanbul, Turkey; vahid@sabanciuniv.edu; 4 Center of Excellence for Functional Surfaces and Interfaces for Nanodiagnostics (EFSUN), Faculty of Engineering and Natural Sciences, Sabanci University, Tuzla, 34956 Istanbul, Turkey; 5 Mechanical Engineering Department, Faculty of Mechanical Engineering, Istanbul Technical University, Gumussuyu, 34437 Istanbul, Turkey; kuddusi@itu.edu.tr

**Keywords:** inertial focusing, microfluidics, spiral microchannels, fluorescent particle separation

## Abstract

Label-free, size-dependent cell-sorting applications based on inertial focusing phenomena have attracted much interest during the last decade. The separation capability heavily depends on the precision of microparticle focusing. In this study, five-loop spiral microchannels with a height of 90 µm and a width of 500 µm are introduced. Unlike their original spiral counterparts, these channels have elliptic configurations of varying initial aspect ratios, namely major axis to minor axis ratios of 3:2, 11:9, 9:11, and 2:3. Accordingly, the curvature of these configurations increases in a curvilinear manner through the channel. The effects of the alternating curvature and channel Reynolds number on the focusing of fluorescent microparticles with sizes of 10 and 20 µm in the prepared suspensions were investigated. At volumetric flow rates between 0.5 and 3.5 mL/min (allowing separation), each channel was tested to collect samples at the designated outlets. Then, these samples were analyzed by counting the particles. These curved channels were capable of separating 20 and 10 µm particles with total yields up to approximately 95% and 90%, respectively. The results exhibited that the level of enrichment and the focusing behavior of the proposed configurations are promising compared to the existing microfluidic channel configurations.

## 1. Introduction

Inertial microfluidics, an emerging tool in scientific studies, offers rapid, continuous, and high-throughput particle focusing and separation and can be mainly utilized in various applications such as blood separation [1], the isolation of cancer cells (Circulating Tumor Cells) [2], disease diagnostics and monitoring [3], and biological processes [4]. For the isolation of targeted particles at the micro scale, a variety of methods have been proposed. These methods are classified into two categories based on the external power requirement: active and passive separation. Many active separation devices, which depend on external forces such as acoustophoresis [5], dielectrophoresis [6,7], magnetic manipulation [8], and optical interference [9], have been developed and tested. Even though these methods provide more accurate results, they have disadvantages of processing small samples at low operating flow rates, complex integration, and expensive process requirements [10]. However, passive techniques such as filtration by sieving structures [11], size and deformability-based trapping [12,13], deterministic lateral displacement (DLD) [14,15], pinched flow fractionation [16], and inertial focusing [2,17,18,19,20,21,22] are implemented by simply exploiting inherent hydrodynamic forces and offer cost-effective and high-throughput alternatives.

Among passive techniques, inertial focusing stands out owing to its simple geometry, easy fabrication, and the capability of qualified cell/particle sorting at high throughput. Spiral microchannels taking the advantage of secondary Dean flows have gained increasing attention. Secondary flows are induced at the transverse cross-section of these devices due to the addition of curvature and Dean drag force so that particles are guided to the corresponding equilibrium positions regarding their size [21,23]. The curvature is introduced into the systems in several forms such as spiral [17,18,22,24], serpentine [25,26], and curvilinear [27] configurations, as well as expansion–contraction structures [28]. The performance of inertial microfluidic devices is highly dependent on the channel geometry, flow conditions, particle size, and particle concentration. The role of the channel shape in Dean-coupled inertial focusing has been extensively studied in the literature. Many types of channel cross-sections such as square [29], rectangular [17,18,22], triangular [30], trapezoidal [31], semicircular [32], and stair-like [33] geometries were investigated. Additionally, the tests with multiple devices working in parallel [34,35,36] were conducted to further increase the throughput. Some research activities focused on the aspect ratio of microchannels [19,22,37]. With the use of the size-dependent hydrodynamic forces, the suspended particles focus into distinctive streamlines along the channel [21,23] and thus the separation performance could be obtained for particles with different sizes.

In this study, spiral sorting devices with modified geometrical parameters were introduced. In the structure of these devices, elliptic configurations were implemented in order to create an alternating radius of curvature along the channels. Normally, regular spiral channels have a linearly increasing radius of curvature, and the intensity of secondary flow decreases gradually. In the proposed devices, the magnitude of Dean vortices varies due to the change in the channel curvature. Yet, the change is not as sudden as in serpentine and curvilinear channels. Due to the alternating curvature, the Dean drag force either increases or decreases along each quarter loop differently from regular spiral channels having an ever-decreasing Dean drag force, which is a key element of particle focusing in curved microchannels. Therefore, the elliptical configurations make particles equilibrate quicker than spiral channels with the help of the changing curvature. Since the lateral velocity profile is not distributed as much as in serpentine channels, the particles also preserve their focusing. Hence, these configurations could lead to an outstanding separation performance.

Four types of devices differing by the initial aspect ratio of elliptic geometry were designed and fabricated. Then, the proposed devices were tested with 10 and 20 μm fluorescent particles at different channel Reynolds numbers to determine the optimum flow rates, at which the best particle separation could be achieved. Later, extensive tests were conducted to assess particle migration and the overall separation yield. 

## 2. Method

### 2.1. Design Principle

**Figure 1:**
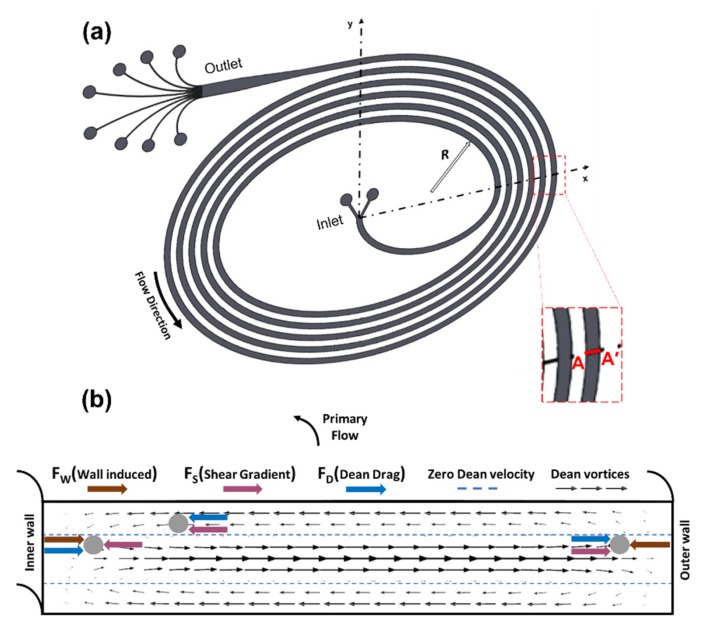
(**a**) Schematic of an elliptic spiral microchannel shown in a Cartesian plane including a radius of curvature, R at a random position, and the location of the A-A’ cross-section. (**b**) A close-up view of the A-A’ plane illustrating secondary flow-induced Dean vortices, zero Dean velocity lines, and a force diagram demonstrating the directions of wall-induced lift force (*F_W_*), shear gradient lift force (*F_S_*), and Dean drag force (*F_D_*) acting on a particle at various locations.

The acting forces on microparticles suspended in a base fluid determine their equilibrium positions. In microchannels with a rectangular cross-section, the inertial lift forces including the shear gradient lift force (*F_S_*) and wall-induced lift force (*F_W_*) are the dominant forces (Figure 1). The parabolic velocity profile stems from Poiseuille flow inside the microchannel and makes microparticles move from the centerline region toward the channel wall, giving rise to the shear gradient lift force. The rotational wake around the particle disappears or an asymmetric wake is generated with a decrease in the distance between the microparticles and walls. As a result, a lift force, known as the wall-induced lift force, directed away from walls toward the channel center appears [21,38]. Accordingly, these lift forces position the particles across the microchannel cross-section at locations, where these forces balance each other so that the focusing positions of particles are established. The net inertial lift force (*F_L_*) resulting from these forces is expressed as:

(1) $$F_{L}=C_{L}\frac{\rho_{f}U_{max}{}^{2}a_{p}{}^{4}}{D_{h}{}^{2}},$$

where $\rho_{f}$ is the fluid density, $U_{max}$ is the maximum fluid velocity, $a_{p}$ is the microparticle diameter, and $D_{h}$ is the hydraulic diameter of the channel. $C_{L}$ is the lift coefficient, which is dependent on the particle position and fluid velocity [21,38]. The magnitude of the $C_{L}$ is zero at the center of the channel and varies between 0.2 and 0.5 in microfluidics applications [27,39].

As a result of curvature addition to the microchannel, secondary flows occur, creating two counter-rotating vortices known as Dean vortices, which lead to the Dean drag force (Figure 1). The following equation includes the Dean drag force, where *µ* and $U_{De}$ represent the fluid viscosity and Dean velocity, respectively [17]:

(2) $$F_{D}=3\pi \mu U_{De}a_{p},$$

where $U_{De}$ is calculated by using the following correlation [40]:

(3) $$U_{De}=1.8\times 10^{-4}De^{1.63},$$

where *De* is defined as the dimensionless Dean number and is given as follows:

(4) $$De=\frac{\rho_{f}U_{f}D_{h}}{\mu }\sqrt{\frac{D_{h}}{2R}}=Re_{C}\sqrt{\frac{D_{h}}{2R}},$$

(5) $$Re_{C}=\frac{\rho_{f}U_{f}D_{h}}{\mu },$$

where *R* is the curvature radius, $U_{f}$ is the mean fluid velocity, and $Re_{C}$ represents the channel Reynolds number. 

The direction of the secondary flow is toward the outer wall around the channel centerline, while the flow recirculates toward the inside wall along the top and bottom regions of the channels, which results in the Dean drag force (*F_D_*) acting on the microparticles, as demonstrated in Figure 1. This force emerges due to the centrifugal acceleration, which moves the fluid at the center faster than the fluid near the top and bottom walls, and thus, the secondary flow formation redistributes the velocity profile and reduces the equilibrium positions of suspended particles to a single one near the inner wall. The Dean drag force and the net inertial lift force manipulate the trajectory of particles in microchannels. Neutrally buoyant particles circulate in two symmetric counter-rotating vortices induced by the pressure difference due to the secondary flow. Near the outer wall, *F_L_* and *F_D_* are in the same direction, and the particles follow the Dean vortices regardless of their size. The particles moving at the top and bottom halves of the microchannel are strongly assisted by lateral Dean flows to migrate to the inward direction. However, the net lift force and Dean drag force on particles act in opposite directions near the inner wall. Regarding the magnitude of these forces, particles will either equilibrate or continue being entrained in the Dean vortex. The ratio of the inertial lift force to the Dean drag force is directly proportional to the third power of the particle diameter (*F_L_*/*F_D_* ∝ *a_p_*^3^) and determines the equilibrium position of the particles. The lift forces dominate by pushing particles to an equilibrium position when *F_L_*/*F_D_* is equal to 1 or greater [20]. 

### 2.2. Microchannel Design

**Figure 2:**
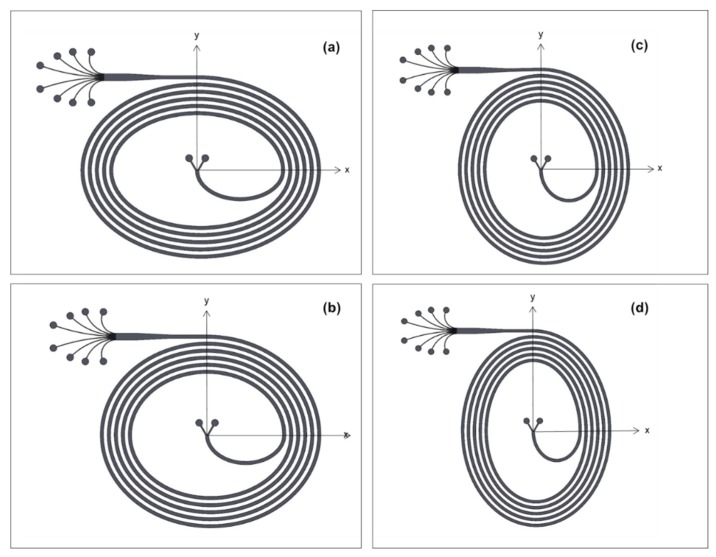
Schematics of the elliptic spiral microchannels: (**a**) Case 1 (initial aspect ratio (IAR): 3:2), (**b**) Case 2 (IAR: 11:9), (**c**) Case 3 (IAR: 9:11) and (**d**) Case 4 (IAR: 2:3).

The ratio of particle diameter to channel hydraulic diameter ratio (*a_p_*/*D_h_*) is reported as a significant parameter for inertial particle migration, and particles can equilibrate across the cross-section of channel due to the effect of Dean flow enabling particles to focus at single streams for *a_p_*/*D_h_* ≥ 0.07 [21,39]. Yet, equilibration in rectangular microchannels depends on the shorter channel dimension, which is the channel height (H), rather than *D_h_* because of shear rate variations across the channel cross-section [17]. In the light of the criterion of *a_p_*/H ≥ 0.07, 500 µm wide and 90 µm high microchannels (Aspect Ratio: 0.18) were fabricated. A five-loop spiral design with two inlets and eight outlets including the prior widened section was considered for sorting 10 µm and 20 µm particles. The length of the microchannels was approximately 43 cm. The distance between two consecutive loops was kept as the same as the channel width. As a novel aspect, elliptic configuration was incorporated in the channel geometry, as shown in Figure 1a. Once the elliptic geometry is introduced as the channel geometry, the intensity of Dean vortices alternates due to the change in centrifugal effects. Hence, the configuration becomes more effective in terms of enhancing the focusing capability. The proposed channels have an initial aspect ratio (IAR) of 3:2, 11:9, 9:11, and 2:3, as seen in Figure 2. With respect to these ratios, four different cases were defined in order to provide a clearer explanation. The initial geometrical parameters for each design are included in Table 1. 

In the Cartesian coordinates as shown in Figure 1a, r_x_ and r_y_ representing the radii of the microchannels on both x and y axes are tabulated in Table 1. Regarding the geometrical parameters of an ellipse, maximum and minimum curvature radii were calculated using the corresponding formulas (Table 1). For Cases 1 and 2 (Figure 2a,b), the maximum curvature radius is located on the y-axis. However, the maximum radius moves to the x-axis in the remaining cases (Figure 2c,d). Moreover, the curvature radius at the outlet of the microchannel changes for each case. Case 1 has the largest radius of curvature at the exit, while the radius of curvature in Case 4 is smaller than the rest (Table 1). 

In a regular spiral channel, the center of the curvature is the same at any point, and the radius increases linearly throughout the microchannel. However, in ellipse-shaped spiral channels, the center of the curvature changes, thereby making the radius of the curvature change along the channel. As a result, the channel curvature radius increases in a curvilinear rising trend. The elliptic design redistributes the intensity of Dean vortices, enabling the focusing patterns to form quickly. Moreover, the outlet radius of the curvature for each case differs due to the elliptic configuration. The Dean number decreases with the increasing radius of curvature at the exit, as the channel shape approaches the horizontal geometry (Case 1 and 2). Contrarily, a smaller curvature radius at the exit results in greater Dean numbers (De) (Case 3 and 4). 

It is also worth noting that Case 1 and Case 4 have similar geometrical features and therefore similar fluid flow characteristics as well as Case 2 and Case 3. The only difference lies in the channel orientation. For instance, the wider part of the microchannel is located in the x-axis for Case 1, whereas the wider part is in the y-axis for Case 4. Even though they have similar shapes, Cases 1 and 2 differ from Case 3 and Case 4 in terms of the final quarter loop configuration, which leads to an increasing or decreasing curvature radius before the straight outlet section. Therefore, this phenomenon will be investigated along with the effect of the changing radius of curvature.

### 2.3. Device Fabrication

The standard soft lithography method was used to fabricate the PDMS (polydimethylsiloxane) microchannels. SU-8 3050 negative photoresist (Microchem Corp., Westborough, MA, USA) was coated on 3” silicon wafers (University Wafer, Inc., Boston, MA, USA). The photoresist was spun at gradually increasing speed to the desired thickness of approximately 90 μm via a spin coater (Dorutek, Ankara, Turkey). The coated Si- afer was exposed to UV light using a Mask Aligner UV-Lithography device (Midas System Co., Ltd., Daejeon, Korea, MDA-60MS Mask Aligner 4”) through the image reversal photomasks printed on acetate (Çözüm Baskı Merkezi, Ankara, Turkey). Then, the photoresist was immersed in a developer (Microchem Corp.) to remove unexposed areas. A PDMS prepolymer base and curing agent (Sylgard 184 silicone elastomer kit, Dow Corning, Midland, MI, USA) were mixed at a 10:1 ratio and then poured over the SU8 master placed in a glass petri dish. The PDMS mixture was degassed for half an hour before curing at 75 °C for 3 h in a vacuum oven (Sheldon Manufacturing, Inc., Cornelius, OR, USA). The baked PDMS was detached from the mold. Inlet and outlet holes were punched using a 21-gauge needle with sharpened tips. Thereafter, both the PDMS channels and 1-mm-thick microscope glass slides were cleaned with isopropyl alcohol and deionized water. After drying with nitrogen gas, surfaces of the glass slide and PDMS channel were activated in an oxygen plasma device (Harrick Plasma Cleaner, Ithaca, NY, USA) with intermittent O_2_ injections for the duration of 1 min. Finally, the enclosed microchannel configurations were formed by pressing the treated surfaces to achieve permanent bonding.

### 2.4. Suspension Preparation

Fluorescently dyed polystyrene microspheres with diameters of 10 µm (Invitrogen), and 20 µm (Fluoresbrite) were used in this size-based separation study. As a medium, deionized (DI) water was utilized to dilute the samples. The dilution concentration of particles in suspension was determined to be lower than 0.01 v/v% for the sake of eliminating particle interactions. The particle suspensions were prepared separately for each size with the help of a magnetic stirring bar in a glass bottle and were loaded to a plastic syringe. Microparticle suspensions were injected through the devices via a syringe pump (Cole-Parmer, Vernon Hills, IL, USA). 

### 2.5. Experimental Setup

The sample of fluorescent polystyrene particles suspended in DI water was injected into the elliptic spiral microchannels through a single inlet using a Cole Parmer syringe pump. Even though two inlets were originally considered in the design, only one inlet was utilized for the current study. The suspension of each particle was pumped into the system at flow rates varying from 0.5 to 3.5 mL/min, which were precisely set by the control unit of the syringe pump. Moreover, a solution containing both 10 µm and 20 µm diameter polystyrene particles was used to check for the compatibility of the results from individual tests. For connection between inlet and outlet openings to the device, TYGON tubing with an internal diameter of 250 µm (LMT-55, IDEX Corp., Harbor, WA, USA) and corresponding fittings (IDEX Corp.,) were utilized. Both videos and image sequences were captured in the expanded outlet section via an inverted phase contrast microscope (Olympus IX72, Olympus, Tokyo, Japan) (equipped with a (12-bit) charge) coupled with a device camera (Olympus DP 72), mercury lamp (Olympus U-LH100HG), and Olympus software (cellSens Imaging Software). The particle migration trajectories were recorded by employing filter cubes of the microscope depending on the dye color of microspheres at exposure time of 600 ms at each flow rate for each case. By injecting a suspension of 10 μm and 20 μm particles, the samples isolated from each outlet were collected into separate small tubes in order to count the number of particles and thus deduce particle separation capability. 

### 2.6. Data Analysis

Along with Olympus software (cellSens), ImageJ(Fiji) software was used for post processing. By analyzing the recorded fluorescent videos, flow fractionation and particle migration were observed at each flow rate. By applying the modules of ImageJ software, the focusing positions and width of focused streamlines for each particle size were determined by stacking discrete frames retrieved from the relevant videos and by investigating the intensity profile across the channel width. Then, the superimposed image of both particles was created by ImageJ software to visualize the capability of multi-particle separation. 

### 2.7. COMSOL Simulations

**Figure 3:**
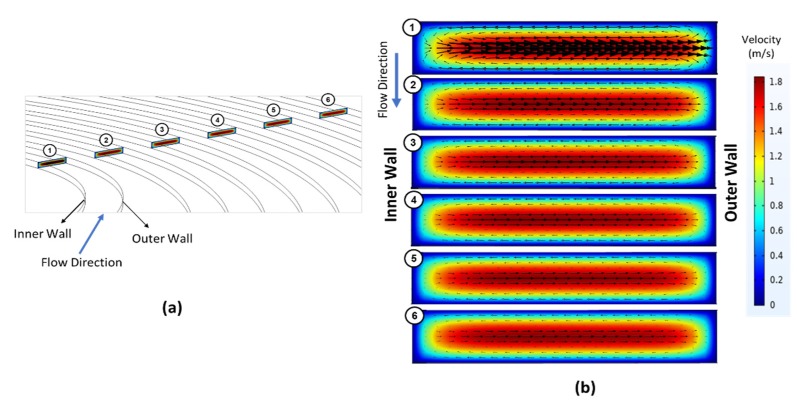
(**a**) Six different cut-plane positions labeled starting from the inner loop along the primary flow direction, and (**b**) a closer look at the vectoral variation of lateral velocity distributions over the colored velocity profile of the primary flow at six different cross-sections throughout the channel. The cross-sectional velocity profiles were retrieved form COMSOL Multiphysics simulation results of Case 1 at volumetric flow rate of 3 mL/min.

The COMSOL Multiphysics 5.5 finite element software program (COMSOL Inc., Stockholm, Sweden) was utilized to simulate Newtonian fluid flows in the four proposed elliptic microchannel configurations. Numerical simulations were done with a computational domain consisting of uniform hexahedral mesh (Figure 3a) with more than 450,000 elements and an average quality of approximately 0.92. These channels were modeled by importing the geometries drawn in 3D (Dassault Systèmes SolidWorks Corporation, Waltham, MA, USA) using mapped meshes, which were refined until the number of elements was optimal. After imposing initial (inlet velocity, outlet pressure) and boundary conditions (no-slip at the walls) properly, each model was solved using the GMRES iterative solver to simulate a single-phase, incompressible, and steady laminar flow governed by the Navier–Stokes equations. The numerical study was conducted for different Reynolds numbers and all the microchannel configurations so that insights about flows in the experimental study could be provided by obtaining Dean vortices in the transverse direction to the channel. Thus, the numerical serves for supporting the experimental findings and for further discussing the effects of velocity distribution on the focusing behavior. 

## 3. Results and Discussion

### 3.1. Laminar Flow Simulation

A computational study was performed in order to understand the underlying fluid flow in the microchannel configurations. Figure 3 demonstrates the variation of tangential velocity vectors over the colored velocity profile of the primary flow at six different positions from the most inner loop to the last one for Case 1. These positions are labeled accordingly in Figure 3a. Figure 3b shows the cross-sectional velocity distribution provided at each cross-section (numbered in Figure 3a, respectively). Accordingly, the Dean vortices in the first loop are more intense and then gradually lose their intensity until the exit of the channel as expected due to the nature of spiral configuration regarding the increasing spiral curvature and Dean number.

**Figure 4:**
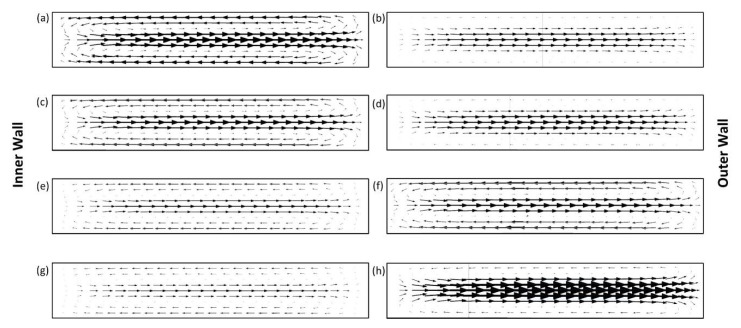
The variations of Dean velocity distribution at the beginning and at end of the last quarter loop of the channel for Case 1 (**a**,**b**), Case 2 (**c**,**d**), Case 3 (**e**,**f**), and Case 4 (**g**,**h**), respectively. The arrow lengths are proportional to the lateral velocity.

Since the microchannels have elliptic configurations, the position of the curve center changes, resulting in the alterations in radius of curvature. Figure 4 illustrates the lateral velocity vectors for all four cases at the beginning and at the end of the last quadrant loop of the channels. The largest Dean velocity can be observed in Figure 4a,h, where the radius of curvature is the smallest. In Case 1 (Figure 2a), the curvature radius decreases throughout the last quarter loop of the channel, and hence the Dean vortices become smaller (from (a) to (b)). This explanation is also valid for Case 4 (Figure 2d) in the exact opposite way. In Case 4, the minimum curvature radius occurs at the exit, leading to a higher lateral velocity (Figure 4h). For Case 2 and Case 3 (Figure 2b,c), where the curvature radius changes within a relatively narrow range, the magnitude of the counter-rotating vortices does not vary drastically along the last ¼ loop prior to the exit, and thus the intensities of the Dean vorticities are rather low.

As Case 1 and Case 4 have similar configurations, the arrow sizes representing the lateral velocity are similar as expected. The variation of curvature radius is greater in Case 1 and Case 4. Figure 4a,h (where the minimum radius is located) illustrates more intense arrows in the middle due to the increased centrifugal effects. Bold arrows show the enhancement of lateral migration, which makes the particles equilibrate faster. Figure 4 also reveals that the intensity of the arrows is less in Case 2 and Case 3 since the change of curvature radius is within a narrower range compared to Case 1 and Case 4. For example, Figure 4a (Case 1) and 4h (Case 4) are expected to exhibit similar velocity distributions regarding the geometric similarity. Since the plane of Figure 4h is located at the end of the curved section of the microchannel, the arrow intensities above and below the zero Dean velocity lines (defined in Figure 1b) start to reduce. This is due to the loss of the Dean drag effect as the flow enters the straight section of the microchannel. The same explanation applies to the rest as well. 

Moreover, the arrows are generally less visible in Figure 4b,d,e,g compared to the rest. The common feature of these subfigures is that they represent the cross-sections, which are located at the end of the quarter loop, where the curvature radius is increasing. The scale of the arrows is smaller at the top and bottom parts of the microchannels since tangential velocities decrease as a result of the increased radius of curvature. These findings lead to less effective migration in the transverse plane.

### 3.2. Lateral Positioning of Particle Streams

**Figure 5:**
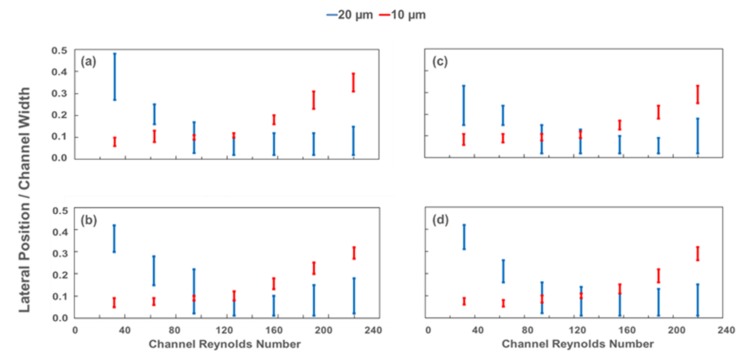
Lateral focusing position from the inside wall normalized by the channel exit width of 1 mm for (**a**) Case 1, (**b**) Case 2, (**c**) Case 3, and (**d**) Case 4 at the outlet.

In this study, elliptic spiral microchannels were employed with the use of 10 µm and 20 µm diameter particles. The particles were tested individually in each channel for several Reynolds numbers. At flow rates within the range of 0.5–3.5 mL/min, fluorescent polystyrene particles diluted in deionized water were introduced into the microchannel through the inlet. For each case, the channel Reynolds number, *Re_c_*, was altered by increasing the flow rate, and the focusing positions of the particle streams in the diverged section prior to the 8-outlet segment were recorded with respect to *Re_c_*. By utilizing the recorded data of both 10-µm and 20-µm fluorescent particle streams at various flow rates, the ratio of lateral focusing positions to channel width at the exit is displayed as a function of the channel Reynolds number in Figure 5. After analyzing these results, the optimum flow rate required to achieve multi-particle separation was determined to investigate the sorting capability for later experiments. The optimum flow conditions assigned for each channel correspond to *Re_c_* values higher than 180 for all the cases. 

**Figure 6:**
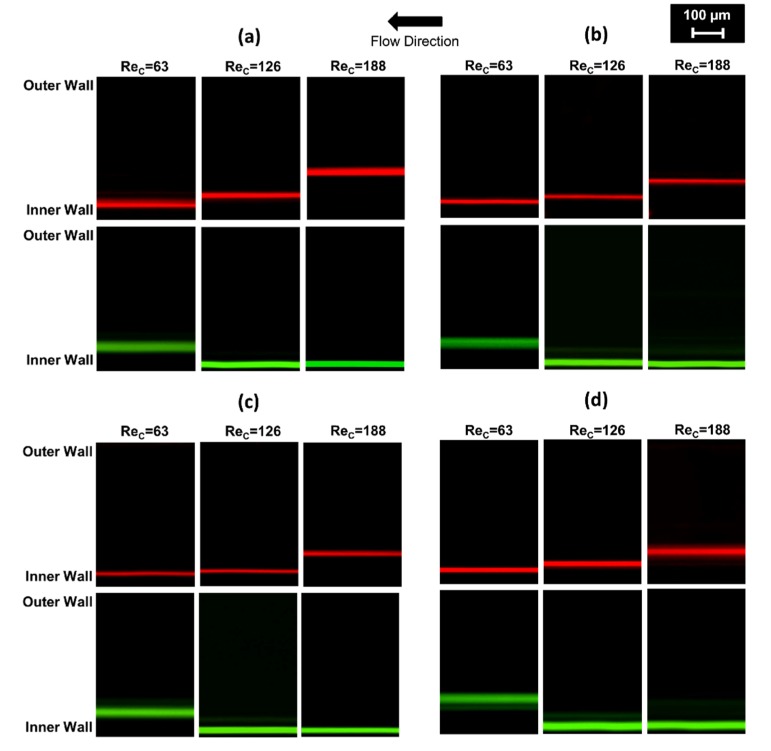
The trajectories of the particle streamlines at the end of the last spiral loop, where the channel width is 500 µm for 10 µm (red lines) and 20 µm (green lines) at *Re_c_* values of 63, 126, and 188 for (**a**) Case 1, (**b**) Case 2, (**c**) Case 3, and (**d**) Case 4.

At lower *Re_c_*, the larger particles are not precisely focused, while smaller particles are located closer to the inner wall. Due to the changing radius in the fabricated elliptic devices, the alternation of the curvature might complicate the flow pattern and disturb the equilibrium of larger particles. Even though the lift force is dominant at lower volumetric flow rates, there is not enough distance in the channel for 20-µm particles to reach equilibrium positions unlike 10-µm particles. Thus, the secondary flow in the radial plane entrains the larger particles and generates distributed streams near the middle of the channel when the *Re_c_* value is smaller than 70. On the other hand, the mixing effects of the Dean drag are surpassed by the lift forces for smaller particles at the smaller flow rates, thereby making them follow the inner wall. The trajectories of the particle streams at the end of the last spiral loop before entering the outlet channel (for each particle size at *Re_c_* of 63, 126, and 188) are demonstrated in Figure 6. 

As 20-µm diameter particles yield higher *a_p_*/*D_h_* ratios, the net lift force acting on the particles causes constant stream positions so that the particles focus within a narrow band of streamlines near the inside wall for Reynolds numbers above 70. Meanwhile, the small particles move from the inner wall to the channel centerline, while large particles migrate contrarily toward the center of the curvature with increasing *Re_c_* values (Figure 5 and Figure 6). As the channel velocity increases, the equilibrium positions of 20-µm diameter particles begin to appear close to the innermost channel wall as a result of the superposition of inertial lift and Dean drag forces. Then, 10-µm particles move toward the channel center with increasing drag force.

Besides, the smaller particles align in considerably narrower streams for each case (Figure 5 and Figure 6). In addition to the intrinsic size difference and non-normalized focus ranges with respect to particle dimension, this finding implies that the smaller particles are easier to be manipulated with the current channel height (aspect ratio). Figure 5 also reveals that both particles display overlapping streams in the middle Rec range (from approximately 70 to 160), which is not suitable for the separation of particles with different sizes.

The lateral focusing positions as a function of the channel Reynolds number in each channel are displayed in Figure 5 and Figure 6. In Cases 1 and 2, where the IAR (initial aspect ratio) > 1, particles with 10-µm diameter remain near the inner wall up to a Reynolds number of approximately 125. Beyond this critical *Re_c_*, the Dean drag force becomes more dominant. Hence, the focusing band of the smaller particles shifts away from the wall. For Cases 3 and 4, where IAR < 1, the critical Reynolds number is slightly higher, above approximately Re 150. This behavior is unexpected, since the curvature radius of the last loop for Cases 1 and 2 is more prone to the horizontal geometry than that of Cases 3 and 4; it is also larger, the Dean number is relatively smaller, and the Dean drag forces are expected to be less effective at the same *Re_c_*. This finding can be explained by the curvature radius change in the last quarter loop. Even though the radius is smaller at the exit for Cases 3 and 4, it decreases significantly along the last ¼ turn. Therefore, the particles leave the last loop before the hydrodynamic forces are fully developed. In Cases 1 and 2, the size of the radius grows to its maximum value in a sudden manner before the entrance of the exit channel. Similarly, undeveloped forces lead to an alteration of the critical Reynolds number range for elliptic channels, which flatten horizontally (Cases 1 and 2) or at the sides (Cases 3 and 4) (Figure 2).

The results emphasize the possibility of separation at lower *Re_c_* due to non-overlapping focusing gaps, where the channel Reynolds number is less than 70. The focusing band is yet larger for 20 µm particles, which makes it difficult to collect samples at a single outlet with the current channel configuration. Besides, the mixing effects of the Dean drag is more dominant, and the streamlines are more dispersed rather than focused, which might cause the particle–particle interaction. Thus, it is preferable to test the devices at higher flow rates. 

**Figure 7:**
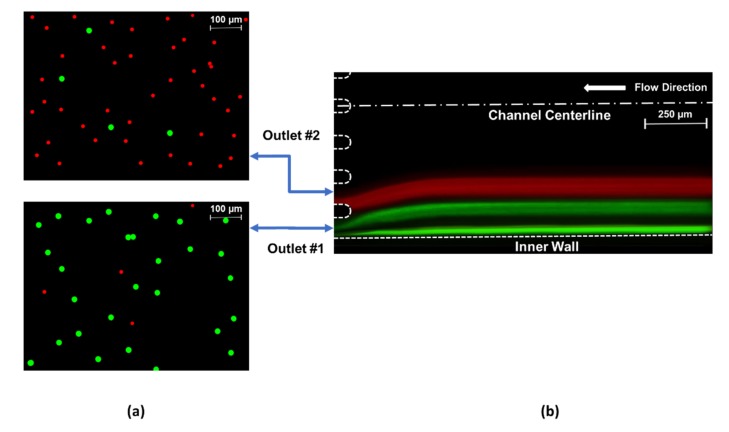
(**a**) The fluorescent microscopic views of samples utilized for particle counting and (**b**) the superimposed fluorescent image of two focused streamlines: red (10 µm) and green (20 µm) illustrating particle migration for Case 4 at the optimum flow conditions (approximately *Re_c_* 195).

From Figure 5, optimal flow conditions are determined for each channel configuration to achieve the best particle separation. These conditions are approximately Re 188 (3.0 mL/min) for Cases 1 and 2 and Re 195 (3.1 mL/min) for Cases 3 and 4. As explained earlier, the optimum flow rate for the separation of 10 µm and 20 µm particles slightly increases for higher De (Dean number) Cases (3 and 4) compared to smaller De Cases (1 and 2). The corresponding Dean numbers for optimal operating conditions are 11, 12.2, 15.4, and 17, respectively, which all meet the criterion of De < 20 for focusing [18,38] and allow the successful sorting of particles with sizes of 10 and 20 µm. Then, 20 µm particles align near the inner wall and are collected by the first outlet tubing, while 10 µm particles focus further from the inner wall and are recovered through the second outlet tubing. Apparently, the particles can be separated into parallel streamlines. The fluorescent images of each particle stream at optimum *Re_c_* values were captured at the channel outlet by utilizing the corresponding filters. The recorded images were superimposed in order to build a composite image showing the two particle streams simultaneously. As a result, Figure 7 was constructed to demonstrate a sample of the separation capability from Case 4 at approximately *Re_c_* 195 (3.1 mL/min). The composite image sections of the collected particle distributions at outlet #1 and outlet #2, which are used in particle counting, can be seen in Figure 7a, while the positions of the focusing streamlines of both 10 µm (red) and 20 µm (green) particles are shown within the channel geometry (Figure 7b).

Figure 6 reveals that the focused streamlines of 10 µm and 20 µm particles are relatively wider for Case 1 and Case 4 (Figure 6a,d), which have similar configurations. The radius of curvature changes in a more intense trend for these cases. This intense variation of curvature radius results in slightly spread streamlines compared to Case 2 and Case 3. On the other hand, thinner streamlines of the focused particles occur in Case 2 and Case 3 (Figure 6b,c), since the curvature radius changes within a relatively narrow range. In addition, 10 µm particles are aligned at locations closer to the channel centerline at Re = 188 for Case 1 and Case 4, where the variation of Dean drag force is larger compared to Case 2 and Case 3. This also emphasizes that the Dean drag forces are more effective regarding the focusing behavior of 10 μm particles at higher flow rates.

Although there is only one focused streamline for 20-µm particles at the end of the last elliptic spiral loop as seen in Figure 6, two focused green streamlines are formed before the eight-branch outlet in Figure 7b. The division of the focused green line is caused by the expanded outlet channel and consequent drop of the aspect ratio. Therefore, the streamlines of both particles are widened compared to Figure 6. Moreover, the equilibrium is also distributed after leaving the curved section of the microchannel, which is due to the disappearance of the Dean drag effects in straight channels, leading to the division of the green streamline. The green streamline adjacent to the inner wall is formed by the fraction of 20 µm particles, which preserves the balance of *F_D_* and *F_L_* throughout the exit. On the other hand, the other pseudo-focused green line consists of the rest of the 20-µm particles. Yet, both green streamlines are recovered from the outlet #1 as aimed.

The spacing between the focusing bands of 10-µm and 20-µm diameter particles is considerably wide at higher flow rate (above approximately *Re_c_* 180). Thus, the width of the microchannel might be contracted to reduce the footprint of the device. On the other hand, this modification causes a larger aspect ratio. Thus, Dean drag effects could become more dominant, which is not desired for separation. In addition, the gap between large and small particles along the lateral direction could allow the separation of particles with closer diameters in the future.

In addition to the channel width, the channel length is another parameter to be investigated. From Asmolov’s lift force equation [41] and Stokes drag, the expression for particle lateral migration velocity is expressed as [39]:

(6) $$U_{L}=\frac{\rho U_{max}{}^{2}a_{p}{}^{3}C_{L}}{3\pi \mu D_{h}{}^{2}},$$

where ρ is the fluid density, μ is the fluid viscosity, $U_{max}$ is the maximum fluid velocity, $a_{p}$ is the microparticle diameter, $D_{h}$ is the hydraulic diameter of the channel, and $C_{L}$ is the lift coefficient. The total channel length required for particles to completely focus at the equilibrium positions is given as:

(7) $$L_{I}=\frac{U_{f}}{U_{L}}\times L_{M},$$

where $U_{f}$ is the fluid mean velocity and $L_{M}$ is the migration length. Similarly, the channel length required for Dean migration is given as [39]:

(8) $$L_{D}=\frac{U_{f}}{U_{De}}\times L_{M}.$$

Using the above equations, the maximum required length for the current work can be calculated as approximately 20 cm. Hence, the current configurations are effective within the range of 0.5–3.5 mL/min. The optimum flow rates achieved in this study are rather high, and thus shorter lengths would be beneficial in terms of reducing the footprint of the device. 

As the base fluid, deionized water, which is a Newtonian fluid, is used in the experiments. Recent studies showed a highly efficient separation by taking advantage of elasto-inertial effects of non-Newtonian fluids [42]. In addition, other investigations confirmed the potential of elastic lift in non-Newtonian fluids for separating particles with a much smaller size [43,44] along with a wider range of flow rates [45,46]. Therefore, the use of viscoelastic fluids will be considered as a future research direction.

### 3.3. Particle Separation Capability

**Figure 8:**
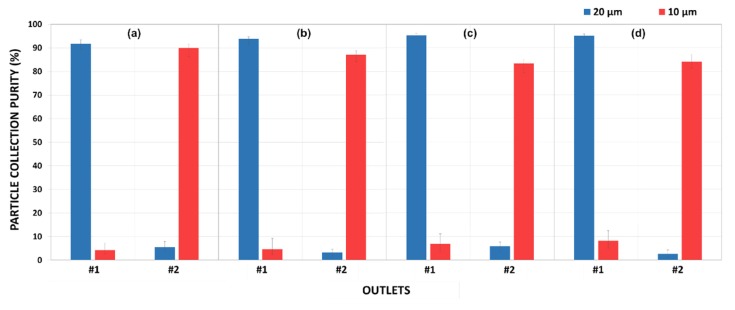
The particle separation purities obtained from the ratio of collected particles at particular outlets to the total collected particles for outlets #1 and #2 in Case 1 (**a**), Case 2 (**b**), Case 3 (**c**), and Case 4 (**d**).

Under optimal conditions, the proposed microfluidic devices were tested until the focused particle streams form. Then, fractions of the streams were collected at all of the eight channel outlets, which were labeled starting from the inner wall. Following that, a particle-counting procedure was conducted for every branch of the outlet. Smaller specimens (as shown in Figure 7a) from each of the collected samples were examined by counting the particles manually with the utilization of the microscope software and ImageJ software tools. This process was repeated several times for the sake of increasing the repeatability of the data. After the amount of particles for each size was counted, the particle collection purities were obtained (Figure 8). There are particles detected in every output fraction. However, the total amount of particles filtered at outlets #1 and #2 covers a significant majority in each case. Therefore, a chart (Figure 8) could be generated only for the first and second outlets. The collected particles from each of the outlets were analyzed to quantify the particle collection purity, which is defined as the ratio of collected particles at particular outlets to the total collected particles. Approximately a 0.01 v/v % volume fraction of particles was considered in the experiments. In addition, the ratio of the amount of the employed particles was 1:1. 

Figure 8 demonstrates that the particle collection purity of larger particles is within the range of 90–95%, while smaller particles are collected with approximately 5% less purity. In addition, the particles filtered out at the outlet #1 and #2 cover 88–98% of the total purity. The maximum collection purities of 95.3% (Case 3) and 90.0% (Case 1) are achieved for 20-µm and 10-µm particles, respectively. Overall, 20-µm particles have a greater purity than 10-µm particles, which can be explained by the more dominant lift force acting on the larger particles at a specified flow rate. Additionally, the Dean drag force is more effective in the focusing regime of the smaller particles under optimum flow conditions. The mixing effects of Dean flow induce relatively more dispersed migration of the smaller particles, which results in less purity.

Overall, all four cases yield a high particle collection purity for large 20-µm particles: 91.9% (Case 1), 93.8% (Case 2), 95.3% (Case 3), and 94.9% (Case 4), with the assistance of the successful focusing of small 10-µm particles further from the inside wall. However, the purity of small 10-µm particles is slightly less than larger particles. The maximum purity is approximately 90.0% for Case 1, while the minimum purity obtained in Case 3 is approximately 83.4%. As *F_D_* increases to the same order of magnitude as *F_L_* for small particles, they are strongly influenced by the Dean drag force in the migration process. Due to the uneven flow distribution at the outlet openings, there is a considerably wider fraction of flow channels into outlet #1 compared to the second one, as seen in Figure 7b. This behavior also deteriorates the purity of the collected 10-µm particles by raising the possibility of small particles to enter the first outlet.

At the optimum flow rates for all cases, the purity of 20-µm particles collected at the first outlet is larger. On the other hand, 10-µm particles can be well collected at outlet #2, yet relatively with less purity, which suggests that the Dean drag forces acting on 10-µm particles are dominant. Thus, small particles are more defocused compared to larger ones. Additionally, the behavior of 20-µm particles is dominated by the lift forces as the Dean velocities are significantly smaller in the proximity of the equilibrium positions/near the sidewalls. Thus, the focused large particles preserve their position. 

Due to the alternating curvature along the last quarter loop of the microchannels, the ratio of *F_D_* at the exit and at the beginning of the last quadrant loop varies for each channel. *F_D_* decreases by nearly half in Case 1, where the exit is more horizontal. This ratio almost doubles in Case 4, where the curvature radius is the smallest among all the cases, which causes a decrease in the purity of 10-µm particles with increasing Dean numbers so that some of the particles exhibit defocusing behavior and migrate to the undesired outlet openings. As the microchannel in Case 1 is less affected by the varying Dean drag force, it yields the highest purity for 10-µm particles. The lift forces dominate in the inertial focusing of 20-µm particles, and the gradual change in Dean flow is thus ineffective in terms of defocusing. Yet, the varying drag force stimulates the non-focusing large particles to shift toward outlet #1, which enhances the purity of large particles with increasing De from Case 1 to Case 4. 

Overall, the proposed devices demonstrate good separation performances for 10-µm and 20-µm particles. Even though Case 3 offers the maximum efficiency (with 95.3%) for 20 µm, among the four cases, Case 1 achieves a purity value above 90% for each particle size. These results indicate that the purity increases more than 5% and 10% for 20-µm and 10-µm particles and the capabilities of elliptic configurations are higher than the reported separation yields for particles with the same sizes [17]. Moreover, these devices also have relatively smaller footprint areas. The minimum footprint area reduction is at least 12% for all the configurations. The footprint area of Case 1 (approximately 11.8 cm^2^), where the best separation is achieved, decreases by more than 20% compared to existing devices [17,31]. In terms of the working velocity (>1 m/s), the proposed configurations enable shorter process times as well when considering the velocities utilized in other studies (<1 m/s) [17,24,33]. In conclusion, it is more advantageous to employ the elliptic configuration with IAR of 3:2, namely Case 1, for separation applications due to the above-mentioned advantages. 

## 4. Conclusions

Different microfluidic devices with elliptic configurations were tested in this study to determine the optimum flow rates to accomplish particle focusing with high purity. These devices can separate suspending particles effectively due to the integration of elliptic geometry into their design, which enhances the rate of lateral particle migration to equilibrium positions and is also proven by large purity values. The results indicate that an inertial sorting of employed microparticles can be succeeded in the proposed microchannel configurations. Purity values are more than 90% for 20-µm particles and 85% for 10-µm particles, which implies that the particles can be effectively recovered at the corresponding outlets. Since a high purity of particle collection is quantitatively obtained for a single process/passage, even higher purity values are possible for a cascade mode (multiple passages). While this study provides valuable insight into the focusing mechanisms and purity performance for cases of spiral microchannels with alternating curvature radius, the scope could be well extended by investigating a variety of particle sizes and further examining inertial focusing dynamics along the whole length of the microchannels. Overall, these devices could be utilized in the high-purity separation of micro-size particles/cells such as CTCs, blood cells, bacteria, viruses, etc. and easily customized for numerous cell/particle-sorting applications in biotechnology.

## Figures and Tables

**Table 1:** The common geometrical parameters and initial geometrical parameters of the first loop for the design of all cases.

	Initial Geometrical Parameters						
	Channel Height (µm)	Channel Width (µm)	r_x_ (mm)	r_y_ (mm)	Initial Aspect Ratio (IRA)	Maximum Radius of Curvature, R_max_ (mm) *	Minimum Radius of Curvature, R_min_ (mm) **
Case 1	90	500	12	8	3:2	18.0	5.3
Case 2			11	9	11:9	13.4	7.4
Case 3			9	11	9:11	13.4	7.4
Case 4			8	12	2:3	18.0	5.3

* R_max_ = max(r_x_^2^/r_y_,r_y_^2^/r_x_) and ** R_min_ = min(r_x_^2^/r_y_,r_y_^2^/r_x_).

## References

[1] Mach A.J., Di Carlo D. (2010). Continuous scalable blood filtration device using inertial microfluidics. Biotechnol. Bioeng..

[2] Ozbey A., Karimzadehkhouei M., Kocaturk N., Bilir S.E., Kutlu O., Gozuacik D., Koşar A. (2019). Inertial focusing of cancer cell lines in curvilinear microchannels. Micro Nano Eng..

[3] Su W., Gao X., Jiang L., Qin J. (2015). Microfluidic platform towards point-of-care diagnostics in infectious diseases. J. Chromatogr. A.

[4] Martel J.M., Smith K.C., Dlamini M., Pletcher K., Yang J., Karabacak M., Haber D.A., Kapur R., Toner M. (2015). Continuous Flow Microfluidic Bioparticle Concentrator. Sci. Rep..

[5] Augustsson P., Magnusson C., Nordin M., Lilja H., Laurell T. (2012). Microfluidic, label-Free enrichment of prostate cancer cells in blood based on acoustophoresis. Anal. Chem..

[6] Zhang C., Khoshmanesh K., Mitchell A., Kalantar-Zadeh K. (2010). Dielectrophoresis for manipulation of micro/nano particles in microfluidic systems. Anal. Bioanal. Chem..

[7] Li Y., Dalton C., Crabtree H.J., Nilsson G., Kaler K.V.I.S. (2007). Continuous dielectrophoretic cell separation microfluidic device. Lab Chip.

[8] Kwak B., Lee J., Lee J., Kim H.S., Kang S., Lee Y. (2018). Spiral shape microfluidic channel for selective isolating of heterogenic circulating tumor cells. Biosens. Bioelectron..

[9] MacDonald M.P., Spalding G.C., Dholakia K. (2003). Microfluidic sorting in an optical lattice. Nature.

[10] Sajeesh P., Sen A.K. (2014). Particle separation and sorting in microfluidic devices: A review. Microfluid. Nanofluidics.

[11] Hosokawa M., Hayata T., Fukuda Y., Arakaki A., Yoshino T., Tanaka T., Matsunaga T. (2010). Size-Selective microcavity array for rapid and efficient detection of circulating tumor cells. Anal. Chem..

[12] Tan S.J., Lakshmi R.L., Chen P., Lim W.-T., Yobas L., Lim C.T. (2010). Versatile label free biochip for the detection of circulating tumor cells from peripheral blood in cancer patients. Biosens. Bioelectron..

[13] Mohamed H., Murray M., Turner J.N., Caggana M. (2009). Isolation of tumor cells using size and deformation. J. Chromatogr. A.

[14] Zeming K.K., Salafi T., Chen C.-H., Zhang Y. (2016). Asymmetrical Deterministic Lateral Displacement Gaps for Dual Functions of Enhanced Separation and Throughput of Red Blood Cells. Sci. Rep..

[15] Liu Z., Huang F., Du J., Shu W., Feng H., Xu X., Chen Y. (2013). Rapid isolation of cancer cells using microfluidic deterministic lateral displacement structure. Biomicrofluidics.

[16] Bhagat A.A.S., Hou H.W., Li L.D., Lim C.T., Han J. (2011). Pinched flow coupled shear-modulated inertial microfluidics for high-Throughput rare blood cell separation. Lab Chip.

[17] Bhagat A.A.S., Kuntaegowdanahalli S.S., Papautsky I. (2009). Inertial microfluidics for continuous particle filtration and extraction. Microfluid. Nanofluidics.

[18] Russom A., Gupta A.K., Nagrath S., Di Carlo D., Edd J.F., Toner M. (2009). Differential inertial focusing of particles in curved low-Aspect-Ratio microchannels. New J. Phys..

[19] Nivedita N., Ligrani P., Papautsky I. (2017). Dean Flow Dynamics in Low-Aspect Ratio Spiral Microchannels. Sci. Rep..

[20] Di Carlo D., Edd J.F., Irimia D., Tompkins R.G., Toner M. (2008). Equilibrium separation and filtration of particles using differential inertial focusing. Anal. Chem..

[21] Martel J.M., Toner M. (2014). Inertial Focusing in Microfluidics. Annu. Rev. Biomed. Eng..

[22] Martel J.M., Toner M. (2012). Inertial focusing dynamics in spiral microchannels. Phys. Fluids.

[23] Di Carlo D. (2009). Inertial microfluidics. Lab Chip.

[24] Lee W.C., Bhagat A.A.S., Huang S., Van Vliet K.J., Han J., Lim C.T. (2011). High-Throughput cell cycle synchronization using inertial forces in spiral microchannels. Lab Chip.

[25] Zhang J., Li W., Li M., Alici G., Nguyen N.T. (2014). Particle inertial focusing and its mechanism in a serpentine microchannel. Microfluid. Nanofluidics.

[26] Di Carlo D., Irimia D., Tompkins R.G., Toner M. (2007). Continuous inertial focusing, ordering, and separation of particles in microchannels. Proc. Natl. Acad. Sci. USA.

[27] Özbey A., Karimzadehkhouei M., Akgönül S., Gozuacik D., Koşar A. (2016). Inertial Focusing of Microparticles in Curvilinear Microchannels. Sci. Rep..

[28] Lee M.G., Choi S., Park J.K. (2011). Inertial separation in a contraction-expansion array microchannel. J. Chromatogr. A.

[29] Choi Y.-S., Seo K.-W., Lee S.-J. (2011). Lateral and cross-Lateral focusing of spherical particles in a square microchannel. Lab Chip.

[30] Mukherjee P., Wang X., Zhou J., Papautsky I. (2019). Single stream inertial focusing in low aspect-Ratio triangular microchannels. Lab Chip.

[31] Guan G., Wu L., Bhagat A.A., Li Z., Chen P.C.Y., Chao S., Ong C.J., Han J. (2013). Spiral microchannel with rectangular and trapezoidal cross-Sections for size based particle separation. Sci. Rep..

[32] Choi J.S., Piao Y., Seo T.S. (2013). Fabrication of a circular PDMS microchannel for constructing a three-Dimensional endothelial cell layer. Bioprocess. Biosyst. Eng..

[33] Ghadami S., Kowsari-Esfahan R., Saidi M.S., Firoozbakhsh K. (2017). Spiral microchannel with stair-Like cross section for size-based particle separation. Microfluid. Nanofluidics.

[34] Sun J., Li M., Liu C., Zhang Y., Liu D., Liu W., Hu G., Jiang X. (2012). Double spiral microchannel for label-Free tumor cell separation and enrichment. Lab Chip.

[35] Warkiani M.E., Khoo B.L., Wu L., Tay A.K.P., Bhagat A.A., Han J., Lim C.T. (2016). Ultra-Fast, label-Free isolation of circulating tumor cells from blood using spiral microfluidics. Nat. Protoc..

[36] Kwon T., Yao R., Hamel J.F.P., Han J. (2018). Continuous removal of small nonviable suspended mammalian cells and debris from bioreactors using inertial microfluidics. Lab Chip.

[37] Zhou J., Giridhar P.V., Kasper S., Papautsky I. (2013). Modulation of aspect ratio for complete separation in an inertial microfluidic channel. Lab Chip.

[38] Di Carlo D., Edd J.F., Humphry K.J., Stone H.A., Toner M. (2009). Particle segregation and dynamics in confined flows. Phys. Rev. Lett..

[39] Bhagat A.A.S., Kuntaegowdanahalli S.S., Papautsky I. (2008). Continuous particle separation in spiral microchannels using dean flows and differential migration. Lab Chip.

[40] Ookawara S., Higashi R., Street D., Ogawa K. (2004). Feasibility study on concentration of slurry and classification of contained particles by microchannel. Chem. Eng. J..

[41] Asmolov E.S. (1999). The inertial lift on a spherical particle in a plane Poiseuille flow at large channel Reynolds number. J. Fluid. Mech..

[42] Nam J., Lim H., Kim D., Jung H., Shin S. (2012). Continuous separation of microparticles in a microfluidic channel via the elasto-Inertial effect of non-Newtonian fluid. Lab Chip.

[43] Lee D.J., Brenner H., Youn J.R., Song Y.S. (2013). Multiplex particle focusing via hydrodynamic force in viscoelastic fluids. Sci. Rep..

[44] Kim J.Y., Ahn S.W., Lee S.S., Kim J.M. (2012). Lateral migration and focusing of colloidal particles and DNA molecules under viscoelastic flow. Lab Chip.

[45] Xiang N., Zhang X., Dai Q., Cheng J., Chen K., Ni Z. (2016). Fundamentals of elasto-inertial particle focusing in curved microfluidic channels. Lab Chip.

[46] Lim E.J., Ober T.J., Edd J.F., Desai S.P., Neal D., Bong K.W., Doyle P.S., McKinley G.H., Toner M. (2014). Inertio-Elastic focusing of bioparticles in microchannels at high throughput. Nat. Commun..

